# Effects of a single bout of low-intensity isometric or moderate-intensity aerobic exercise on postexercise hypotension in adults of African and south-Asian descent: a randomized controlled crossover trial

**DOI:** 10.3389/fresc.2026.1882762

**Published:** 2026-07-31

**Authors:** Marina Bersaoui, Se-sergio Baldew, Karla Goessler, Kwame Van der Hilst, Gail Oldenstam, Pooja Chote, Shreya Basgiet, Jerry Toelsie, Veronique Cornelissen

**Affiliations:** 1 Department of Physical Therapy, Discipline Rehabilitation for Internal Disorders, Faculty of Medical Sciences, Anton de Kom University of Suriname, Paramaribo, Suriname; 2 Department of Rehabilitation Sciences, Cardiovascular Exercise Physiology Unit, KU Leuven, Leuven, Belgium; 3 Cruzeiro do Sul Virtual Group, University City of Sao Paulo, Sao Paulo, Brazil; 4 Department of Cardiology, Academic Hospital of Paramaribo, Paramaribo, Suriname; 5 Department of Physiology, Faculty of Medical Sciences, Anton de Kom University of Suriname, Paramaribo, Suriname

**Keywords:** aerobic exercise, blood pressure, ethnicity, hypertension, isometric exercise, post-exercise hypotension

## Abstract

**Introduction:**

Current guidelines on physical activity and exercise to manage blood pressure (BP) in adults with hypertension are predominantly based on studies performed in Caucasian populations and little is known about the potency of exercise as a BP-lowering tool in non-Caucasians. Therefore, the “Hypertension, Ethnicity and Exercise” (HYPE^2^X) trial aimed to investigate whether a short bout of aerobic exercise or isometric strength exercise lowers BP in a non-Caucasian population.

**Methods:**

African and South-Asian descendants living in Suriname (18–65 years) with high-normal BP or grade I hypertension were invited to participate in a randomized controlled crossover trial. Participants completed: a 30-minute aerobic exercise (AEX) treadmill walking/running bout at 40%–60% of heart rate reserve, an isometric handgrip (IHG) session at 30% of maximal handgrip strength and a sitting control session. Office BP was measured 30 min after the experiment and ambulatory mean 8-hours, daytime and nighttime BP. Statistical analyses were performed using linear mixed models.

**Results:**

A total of 29 adults of African descent [mean age: 48.7 ± 9.9 years, 31% male, mean systolic BP (SBP) and diastolic BP (DBP) were 142.9 ± 11.2 and 88 ± 8.0 mmHg] and 28 adults of South-Asian descent (mean age: 51.5 ± 10.6 years, 46.4% male, mean SBP: 138.2 ± 6.9 and DBP 85.7 ± 4.5 mmHg) were included. In Africans, systolic BP was significantly reduced after AE at post 30 min [−9.0, 95%CI: (−13.4, −4.7) mmHg, *p* < 0.001], non-significantly reduced during daytime [−4.08, 95%CI (−0.26, −7.75) mmHg, *p* = 0.08]. Diastolic BP was non-significantly reduced after AEX at post 30 min [−3.46, 95%CI: (−6.56, −0.36) mmHg, *p* = 0.08] and non-significantly reduced during daytime hours [−3.07, 95%CI: (−0.27, −5.90) mmHg, *p* = 0.08]. In South-Asians systolic and diastolic BP were significantly reduced after AE at post 30 min [−7.40, 95%CI [−12.2, −2.6] mmHg, *p* < 0.01/−4.4, 95%CI [−7.5, −1.3] mmHg, *p* = 0.01].

**Conclusion:**

A single bout of AEX resulted in an immediate reduction in office BP at 30-minutes postexercise in both the African and South-Asian groups, while a more favorable reduction in daytime BP was observed in the African group only. A single bout of IHG did not elicit PEH in either ethnicity. Further exploration is needed to assess different intensity levels, volumes, or frequency levels of exercise in non-Caucasian populations.

## Introduction

Hypertension continues to be the most prevalent cardiovascular (CV) risk factor in the world, and according to global estimates its prevalence has increased from approximately 650 million adults in 1990 to 1.28 billion adults worldwide, of which two-thirds live in low- and middle-income countries, including African and Asian populations (1, 2). It is the leading risk factor for global mortality, with approximately 13% of all deaths attributable to hypertension (1, 2). Despite its huge impact on public health and the costs of health care, only 42% of individuals with hypertension receive treatment, and only 21% have their hypertension controlled (2). The higher prevalence of hypertension in African and Asian populations appears to be multi-factorial and can be attributed to social health disparities, limiting access to health care, urbanization, unhealthy diet, physical inactivity, or genetic predisposition, which all have been associated with higher BP levels and poorer BP control (3–5). Therefore, establishing effective, adherable, non-pharmacological approaches implementable worldwide may prove pivotal in tackling the global hypertension crisis.

Acute exercise is known to elicit immediate BP reductions of approximately 5–8 mmHg, which may be sustained for up to 24 h after completion of the session (6). This exercise-mediated reduction in BP, which helps patients with elevated BP or hypertension maintain their BP within a more normal range for a specific period of the day, is referred to as postexercise hypotension (PEH) (7, 8). PEH has been consistently documented after a single session of aerobic endurance exercise with BP reductions being more pronounced in individuals with higher baseline BP and after higher intensity or larger duration of exercise sessions (9–11). Additionally, a session of dynamic resistance exercise has also been shown to elicit PEH, with reported systolic BP reductions ranging between approximately 3–6 mmHg depending on exercise volumes and intensity (11, 12). Therefore, guidelines recommend daily aerobic or dynamic resistance exercise as a key lifestyle intervention in the management of hypertension (13–15). Yet, although PEH has been widely studied in Caucasian populations (15), results from our recent meta-analysis underscored the lack of robust randomized controlled trials (RCT) evaluating the existence of PEH in patients of Asian or African descent (16). Evidence from nine studies (4 in African and 5 in Asian descendants) suggested that a single session of aerobic exercise did not significantly reduce BP in these non-Caucasian populations, neither at 30 min post-exercise nor during 24-hour ambulatory BP monitoring (16). These findings indicate that the presence and magnitude of PEH may differ across ethnic populations and support the need for further controlled studies in non-Caucasian populations.

Furthermore, rapidly accumulating evidence suggests that regular isometric exercises, such as wall squats or handgrip exercises lead to BP reductions that are at least equally effective as those achieved with guideline recommended aerobic endurance or dynamic resistance exercises (17, 18). Meta-analyses report reductions in resting systolic BP of approximately 5–10 mmHg and diastolic BP of 3–6 mmHg following isometric training interventions (18, 19). Therefore, isometric exercises, which are easily accessible at no cost hold significant potential as a BP-lowering intervention for a large group of patients worldwide. However, data on the immediate response of BP following isometric exercise remains inconsistent across studies (19). Previous evidence indicates that a single bout of isometric exercise may not result in a significant reduction in BP, whereas repeated isometric training interventions have been shown to reduce systolic BP by approximately 5–10 mmHg and diastolic BP by 3–6 mmHg (19).

Therefore, the main aim of this study was to investigate the acute effects of a single bout of isometric handgrip exercise (IHG) and a single bout of aerobic endurance exercise (AEX) on daytime BP during the subsequent activities of daily living in patients with high-normal to grade 1 hypertension of South-Asian or African descent living in Suriname. Secondary outcomes included office BP measured 30 min post-intervention, mean nighttime BP, mean 24-hour ambulatory BP, and hourly ambulatory BP responses during the first 8 h following the intervention.

## Methodology

### Study design and setting

The “Hypertension, Ethnicity and Exercise” (HYPE^2^X) trial was a randomized controlled cross-over trial conducted at the Anton de Kom University of Suriname (Paramaribo, Suriname), evaluating the effect of a single bout of AEX or IHG on ambulatory BP (ABP) and BP related mechanisms in healthy adult men and women of African or South-Asian descent with high-normal BP or grade I hypertension. Participants were classified as belonging to either the African or South-Asian ethnic group if at least three of their grandparents were of the respective ethnicity (20).

The study protocol was approved by the Ethical Committee of the Ministry of Health of Suriname on the 29th of January 2020 (ref. no. 183/2020), and the study was conducted according to the principles of the World Medical Association Declaration of Helsinki on ethnics in medical research. The trial is reported in accordance with the Consolidated Standards of Reporting Trial guidelines (21). The trial was registered at clinical trials.gov: NCT06824805. Written informed consent was obtained from all participants before enrollment in the study.

### Recruitment and screening

Participants were eligible if they were between 18 and 65 years of age, of African or South-Asian descent, insufficiently physically active according to the WHO physical activity recommendations based on self-report, non-smokers, and either untreated or treated with a maximum of two antihypertensive medications. Participants were excluded if they had functional or cognitive limitations affecting the correct performance of the exercise intervention, a history of ischemic stroke, autonomic neuropathy, cardiovascular, renal, or pulmonary disease, diabetes mellitus type I or II, pregnancy, exercise-induced signs of myocardial ischemia or complex ventricular arrhythmias during CPET, resistant hypertension, or use of three or more antihypertensive medications.

Potential candidates were recruited via advertisement at outpatient clinics, social media webpages and via flyers. During the screening, office BP was measured in triplicate after a 10-min seated rest on two separate days (visit 1 and visit 2) to confirm the diagnosis of high normal BP or hypertension, in accordance to the ESH guidelines (14).

Participants meeting the BP eligibility criteria (i.e., systolic BP: 130–159 mmHg and/or diastolic BP: 85–99 mmHg) were then invited for a third visit which included completing a standardized health questionnaire, a cardiovascular risk assessment using the American College of Sports Medicine (ACSM) risk stratification questionnaire (22), the Global Physical Activity Questionnaire (GPAQ) (23) and the WHO Salt Intake Questionnaire (24) followed by an assessment of anthropometrics and a maximal cardiopulmonary exercise test (CPET).

#### Anthropometric characteristics

Body length and weight were measured using a Seca 213 stadiometer (Seca GmbH & Co. KG, Germany) and a Seca750 analog scale (Seca GmbH & Co. KG, Germany). The waist circumference was measured with a Seca 210 stretch resistant measuring tape (Seca GmbH & Co. KG, Germany) by placing it between the last rib and the iliac crest and measurement taken at the end of a normal expiration. Body mass index was calculated as the ratio of weight (kg)/length (m)^2^.

#### Cardiopulmonary exercise test

Exercise capacity and cardiovascular responses during exercise were assessed during a graded symptom limited CPET on a cycle ergometer. Twelve-lead ECG (Cardiosoft, GE Healthcare, UK), ventilation and gas exchange measurements (Oxycon Pro, JaegerTM, CareFusion, Germany) were recorded continuously during the test and for three minutes into recovery. Initial workload was set at 40 watts followed by increments of 20 watts every minute until volitional exhaustion. In and expired gasses were analyzed breath-by-breath by means of the Oxycon Pro. The test was considered maximal when the participant reached a respiratory exchange ratio >1.1 (25). Peak oxygen uptake (VO_2_ peak) was defined as the highest 30-s average of VO_2_ obtained during the test. The percentage of predicted peak VO_2_ was calculated using the references values of Wasserman et al. (25). Resting HR and peak HR were used to calculate the training HR for the aerobic exercise session.

### Experimental sessions

After the screening period, participants attended the laboratory on three separate occasions to complete three experimental sessions in a randomized order in a temperature-controlled room: an aerobic exercise session, an isometric handgrip exercise session, and a control session. Randomization was performed using sealed envelopes containing the intervention conditions. At the first intervention visit, participants drew one of three lots (AEX, IHG, or control) to determine the intervention for that session. At the second intervention visit, participants drew one of the two remaining lots to determine the second intervention, while the remaining intervention condition was performed at the third visit. Neither the participant nor the researcher knew which intervention would be performed until the lot was drawn on the study day. Due to the nature of the exercise interventions, participant and outcome assessor blinding was not feasible. All tests were performed in the morning (7:00–10:00 or 10:00–13:00) at the same time of day for each individual patient to control for diurnal variation in BP and to avoid any influence of time of medication intake. Between the experimental sessions there was a wash-out period of minimal 7 days.

**Figure 1:**
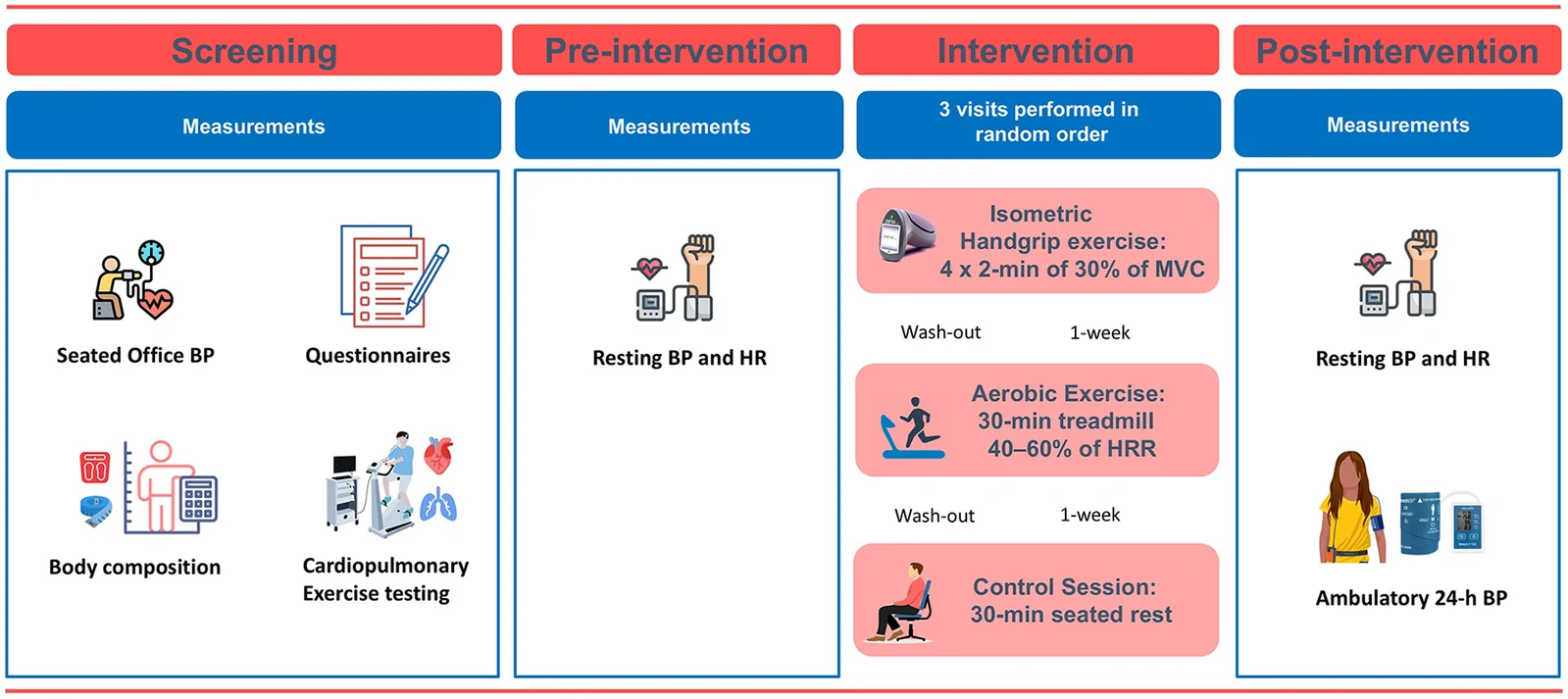
Experimental study overview. BP, blood pressure; HR, heart rate; MVC, maximal voluntary contraction; HRR, heart rate reserve.

Figure 1 shows an overview of the flow of the experimental sessions. Each session started with 1) a pre-intervention assessment of BP and HR after a 10-minute supine rest, followed by 2) an intervention, 3) a post intervention assessment and ended with 4) a 24-hour ABP measurement and completion of a physical activity diary in the home environment.

#### Acute aerobic session

Participants performed 30 min of aerobic exercise on a treadmill at moderate intensity corresponding to a HR ranging between 40%–60% of HR reserve (HRR) (26). Compliance to the training intensity was monitored using ECG.

#### Acute isometric exercise session

Participants performed a standardized bilateral isometric handgrip protocol (27) consisting of four sustained contractions of 2-minutes at a maximal voluntary contraction of 30% using a programmed digital hand dynamometer (ZonaPlus, Zonahealth, Boise, id, uSa). Each set of a 2-minute contraction is followed by a 1-minute resting period. Before the start of a session, a maximal voluntary contraction (MVC) was performed with each hand. During the session, the participants received visual and auditory feedback to ensure compliance to the IHG protocol. Compliance to the IHG protocol was defined as achieving a score of at least 85%.

#### Control session

The control session consisted of 30-minutes of seated rest in a quiet room. The patients were allowed to read while seated.

#### Outcomes measurements

The primary outcome of this study was mean ambulatory daytime BP, defined as the average BP during the first 8 h following completion of the experimental session. Secondary outcomes included office BP measured 30 min post-intervention, mean nighttime BP, mean 24-hour ambulatory BP, and hourly ambulatory BP responses during the first 8 h following the intervention.

#### Office blood pressure

Before each experimental session, BP and HR were assessed in triplicate, after a 10-minute supine rest period, using an automated ambulatory 24-hour BP device, Watch BP O3 (Microlife, Widnau, Switzerland). The mean of these three readings was used as the pre-intervention BP. After each experimental session, participants were repositioned in the supine position and BP was measured 30-minutes after the session.

#### Ambulatory blood pressure monitoring

Subsequently, the participants left the laboratory wearing the ABP monitor (Watch BP 03; Microlife, Widnau, Switzerland) on the non-dominant arm for 24-hours. The ABP monitor was programmed to measure BP every 30 min. Participants were instructed to proceed with their normal daily activities and were asked to fill in an activity diary. Daytime BP was defined as the average BP over a period of 8 h after completion of the experimental session. The first 8-hours post-intervention were chosen to ensure that BP measurements reflected waking hours for all participants, irrespective of the time at which the intervention was performed. Nighttime BP was defined based on the reported sleeping and awakening hours. An ABP data file was included in the analysis if there was at least one valid reading per hour and at least 70% of the readings were successful. Therefore, each ABP file was manually evaluated to detect invalid measurements. Measurements were defined as invalid when SBP > 240 mmHg or<50 mmHg, or DBP > 140 mmHg or <40 mmHg and in the presence of a BP deviation of ±50 mmHg for SBP and ±20 mmHg for DBP between two successive measurements (28), which could not be explained based on the activity diary.

### Sample size

An *a priori* power analysis (G*Power version 3.1.9.4; Heinrich Heine University Dusseldorf, Dusseldorf Germany) showed that for an expected reduction in office SBP of −7 ± 4 mmHg (29) following aerobic exercise and a reduction of −5.4 ± 7.3 mmHg following IHG (27) compared to control intervention, we would need a minimum of 20 study participants in an aerobic exercise and 23 study participants for isometric handgrip exercise (powe*r* = 0.95, alfa = 0.01). As the primary objective of the study was to evaluate exercise-induced BP changes within adults of African descent and within adults of South-Asian descent, the sample size calculation was based on the expected intervention effects and not on direct comparisons between ethnic groups.

### Statistical analysis

All statistical analyses were performed using the software programme, Jeffreys's Amazing Statistics Program (JASP, Version 0.16.2.0). Continuous data are reported as mean ± standard deviation (SD) or standard error (SE) or mean confidence intervals (CI: 95%) with statistical significance set at a two-tailed *p*-value < 0.05. Comparison of baseline demographic and clinical characteristics of the African and Asian group was performed using a two-tailed unpaired student *t*-test or chi-square. Assumptions of normality were evaluated using the Shapiro–Wilk test and Q-Q-plot. We used linear mixed models to analyze data to assess PEH. We included postexercise BP (i.e., office post-30 min BP, average daytime BP and average nighttime BP) as the dependent variable, type of intervention as fixed effect and the pre-exercise BP as covariate variables. Additional sensitivity analyses were performed including antihypertensive medication use (medicated vs. non-medicated) and calcium channel blocker use (yes/no) as covariates in the mixed models. We also measured the BP response to exercise training by means BP of hourly intervals for 8 h post exercise vs. the day of the resting control session with intervention and time as repeated factors.

## Results

### Participants

**Figure 2:**
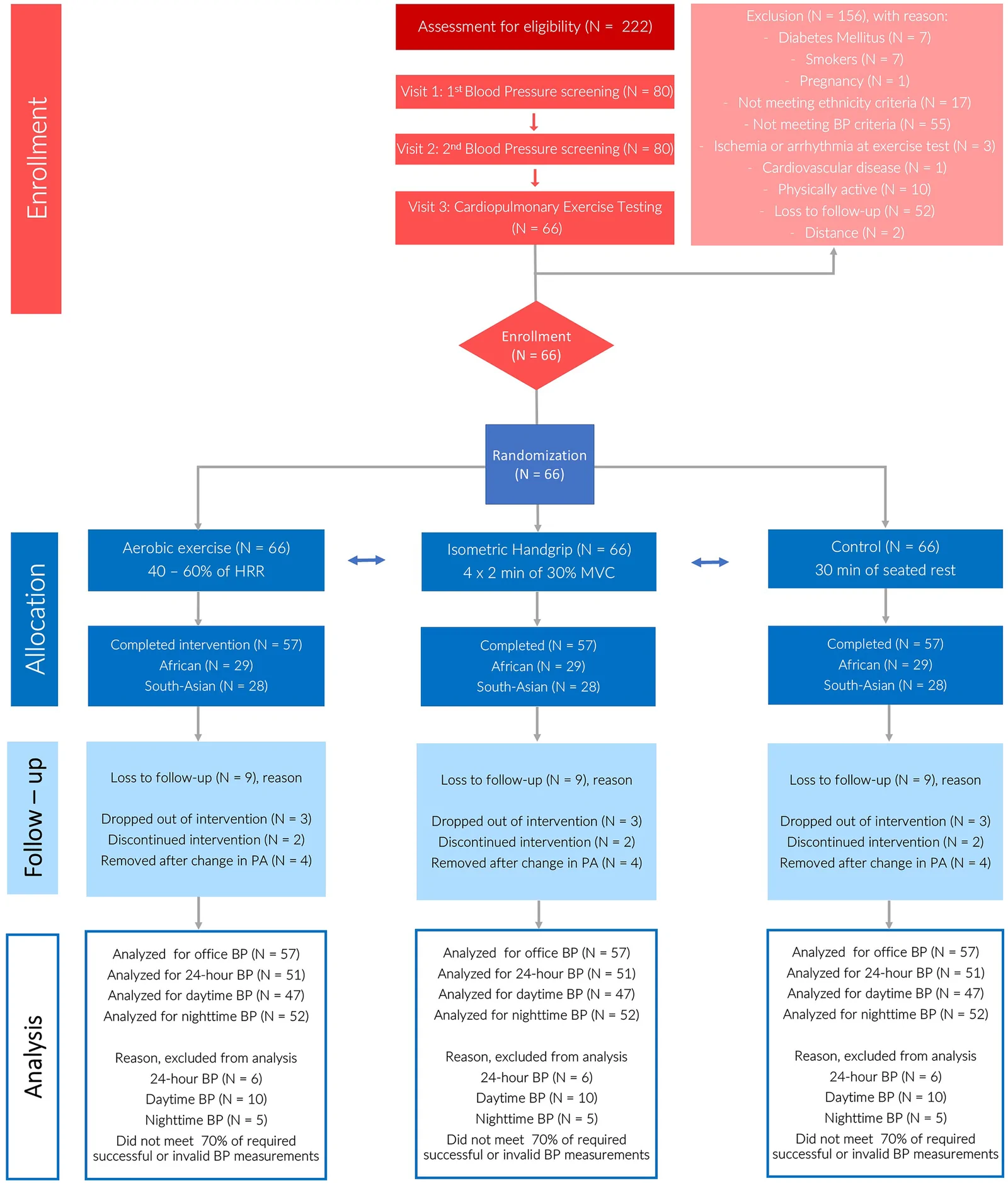
Study design, CONSORT chart.

An overview of the study flow is shown in Figure 2. Two hundred and twenty-two individuals (*n* = 116 Africans; *n* = 106 Asians) volunteered for participation of which 66 (*n* = 34 Africans; *n* = 32 Asians) were eligible for inclusion. Three participants immediately withdrew after randomization due to personal reasons (i.e., lack of time, busy work schedule, did not attend the session) and one participant dropped out after the first experimental session due to lack of time. Data of four participants (*n* = 2 Africans, *n* = 2 Asians) was excluded due to a change in their habitual level of physical activity during the intervention period. One participant was excluded for not completing the control session (*n* = 1 African). In total, fifty-seven participants (*n* = 29 Africans; *n* = 28 Asians) were included and completed all experimental sessions.

Baseline characteristics of the participants included in the final analysis are presented in Table 1. The majority of the participants were middle-aged, obese, medicated and physically unfit for their age, with no significant differences between both ethnic groups with the exception of a higher usage of calcium-channel blockers (CBBs) in the African descents compared to the Asian descents (*P* < 0.01). No adverse events, cardiovascular complications or exercise-related injuries occurred during any of the experimental sessions.

**Table 1:** Characteristics of the study population.

Characteristics	Total sample	African descents	Asian descents	*P*-value
	(*N* = 57)	(*N* = 29)	(*N* = 28)	
Age (years)	50.1 ± 10.3	48.7 ± 9.9	51.5 ± 10.6	0.31
Sex, % male (*n*)	39 (22)	31 (9)	46 (13)	0.23
Body mass (kg)	88.6 ± 19.6	88.8 ± 20.3	88.3 ± 19.3	0.91
BMI (kg/m^2^)	31.7 ± 5.9	31.5 ± 5.4	31.8 ± 6.5	0.85
Waist circumference (cm)	100 ± 18	96 ± 14	104 ± 20	0.10
Office systolic BP (mmHg)	141 ± 10	143 ± 11	138 ± 7	0.13
Office diastolic BP (mmHg)	87 ± 7	88 ± 8	86 ± 5	0.19
Peak VO2 (mL/kg/min)	16.9 ± 4.6	16.3 ± 4.1	17.5 ± 5.1	0.24
Wasserman predicted %		70.0%	75.6	
RER	1.2 ± 0.1	1.2 ± 0.1	1.2 ± 0.1	0.46
Moderate, PA level,				
No MPA, % (*n*)	60 (34)	65 (19)	53 (15)	0.39
Somewhat MPA, % (*n*)	40 (23)	35 (10)	47 (13)	
Mean minutes/week	31.3 ± 45.4	26.3 ± 41.6	36.6 ± 49.2	
Vigorous, PA level,				
No VPA, % (*n*)	88 (50)	87 (*N* = 25)	90 (*N* = 25)	0.86
Somewhat VPA, % (n)	12 (7)	13 (*N* = 4)	10 (*N* = 3)	
Mean minutes/week	6.5 ± 16.0	6.1 ± 9	6.9 ± 20.9	
Moderate to-vigorous, PA level				
No MVPA, % (*n*)	56 (32)	61 (*N* = 17)	52(*N* = 15)	0.40
Somewhat MVPA, % (*n*)	44 (25)	39 (*N* = 11)	48 (*N* = 14)	
Mean minutes/week	37.8 ± 49.7	32.3 ± 45.3	43.4 ± 54.2	
Categories of hypertension:				
High-normal blood pressure, % (*n*)	58 (33)	62 (29)	53 (28)	0.52
Hypertension grade I (%)	42 (24)	38 (29)	47 (28)	0.52
Medicated (%)	81 (46)	86 (25)	75 (21)	0.70
Type of antihypertensive medication				
ACE inhibitors	23 (13)	14 (4)	32 (9)	0.10
Beta-blockers	30 (17)	28 (8)	32 (9)	0.71
Calcium channel blockers	46 (26)	66 (19)	25 (7)	0.002*
Diuretics	18 (10)	21 (6)	14 (4)	0.53
Angiotensin receptor blockers	12 (7)	14 (4)	11 (3)	0.72

Data are reported as means ± S.D.

* *p* < 0.05.

### Exercise data

All participants performed aerobic exercise within the prescribed HR training zone, defined as moderate intensity (i.e., 40%–60% of HRR) (26). Specifically, the mean training HR for participants of African descent was 118.8 ± 10.8 bpm (51.2% of HRR), while participants of Asian descent had a mean HR during the aerobic session of 116.4 ± 12.9 bpm (51.4% of HRR). The performance score for IHG was 90.5 ± 5.64% for participants of African descent and 90.2 ± 7.29% for participants of Asian descent, with no significant differences observed between the groups (*p* > 0.05).

### Postexercise hypotension: office blood pressure

**Figure 3:**
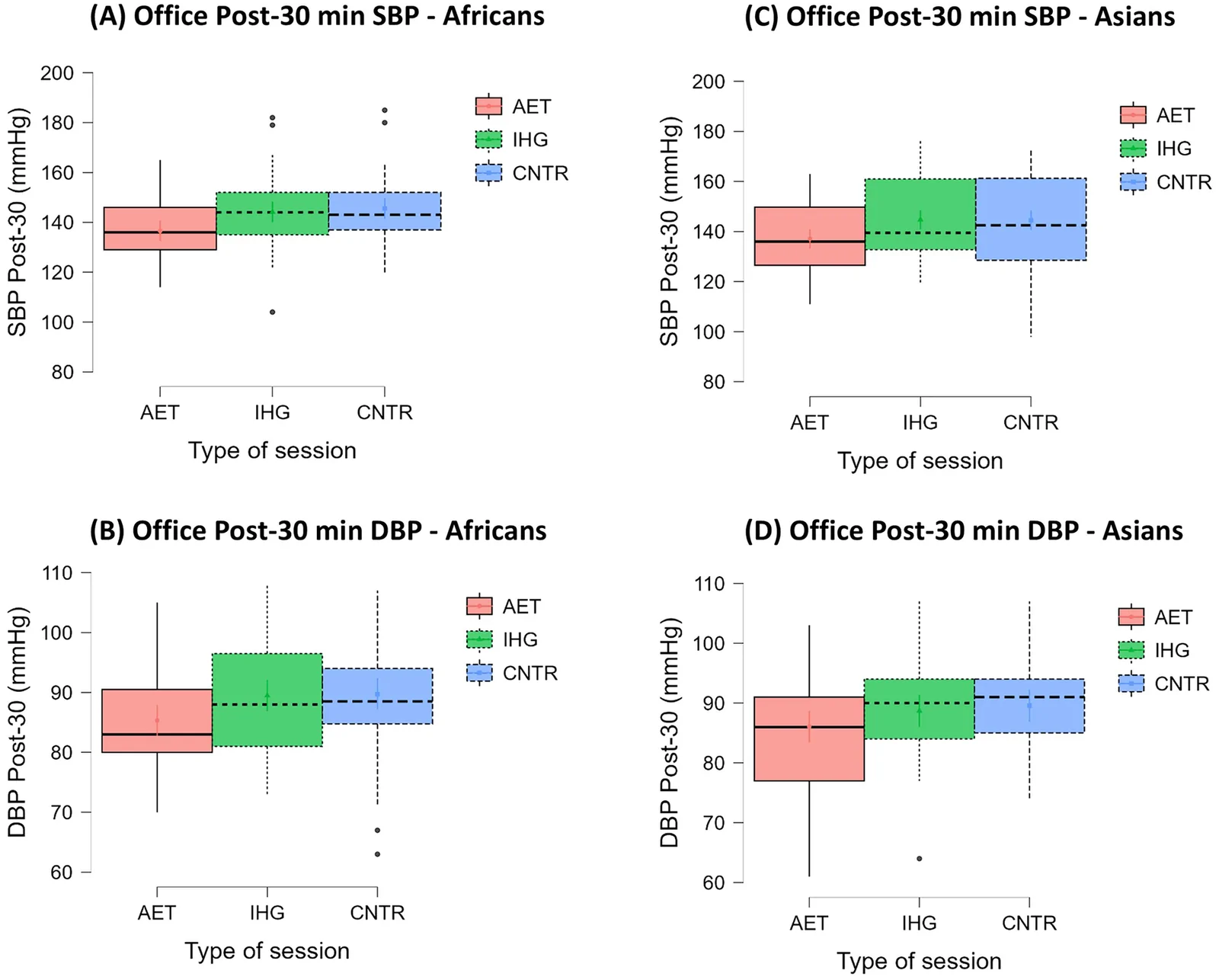
**(A–D)** systolic and diastolic BP at 30 min after the aerobic, isometric exercise or control sessions of the African group (*N* = 29) are presented in **(A)** and **(B)** and of the asians (*n* = 28) in **(C)** and **(D)**; * = *p* < 0.05 compared to control.

As shown in Figures 3A–D, a single bout of aerobic exercise resulted in a reduction in systolic and diastolic BP at 30 min postexercise in individuals of African descent [−9.03, 95% CI: [−13.35, −4.71] mmHg, *p* < 0.001/95% CI: −3.46 [−6.56, −0.36] mmHg, *p* = 0.08] and individuals of Asian descent [−7.40, 95% CI: [−12.19, −2.61] mmHg, *p* < 0.01/−4.41, 95% CI: [−7.48, −1.33] mmHg, *p* = 0.01] compared to the control session. A single bout of isometric handgrip exercise had no effect on systolic or diastolic BP at 30 min postexercise in the individuals of African [−1.42, 95% CI: [−5.72, 2.89] mmHg, *p* = 0.51/−0.82, 95% CI: [−3.93, 2.31] mmHg, *p* = 0.61] or Asian descent [+0.20, 95% CI: [−4.60, 4.99] mmHg, *p* = 0.93/−0.25, 95% CI: [−3.33, 2.81] mmHg, *p* = 0.87] compared to the control session. Detailed information can be found in Supplementary Tables S1 and S2.

### Postexercise hypotension: ambulatory blood pressure

**Figure 4:**
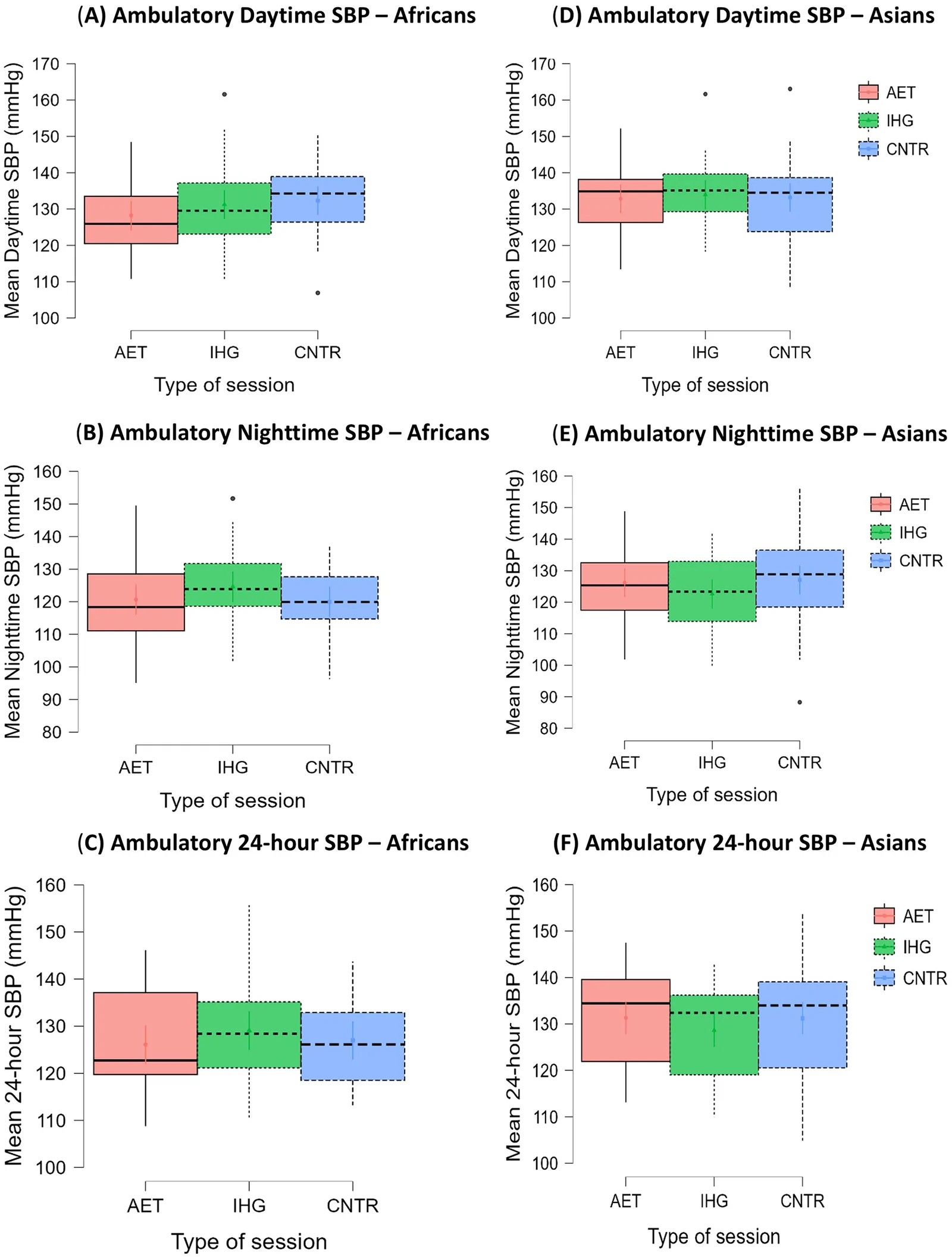

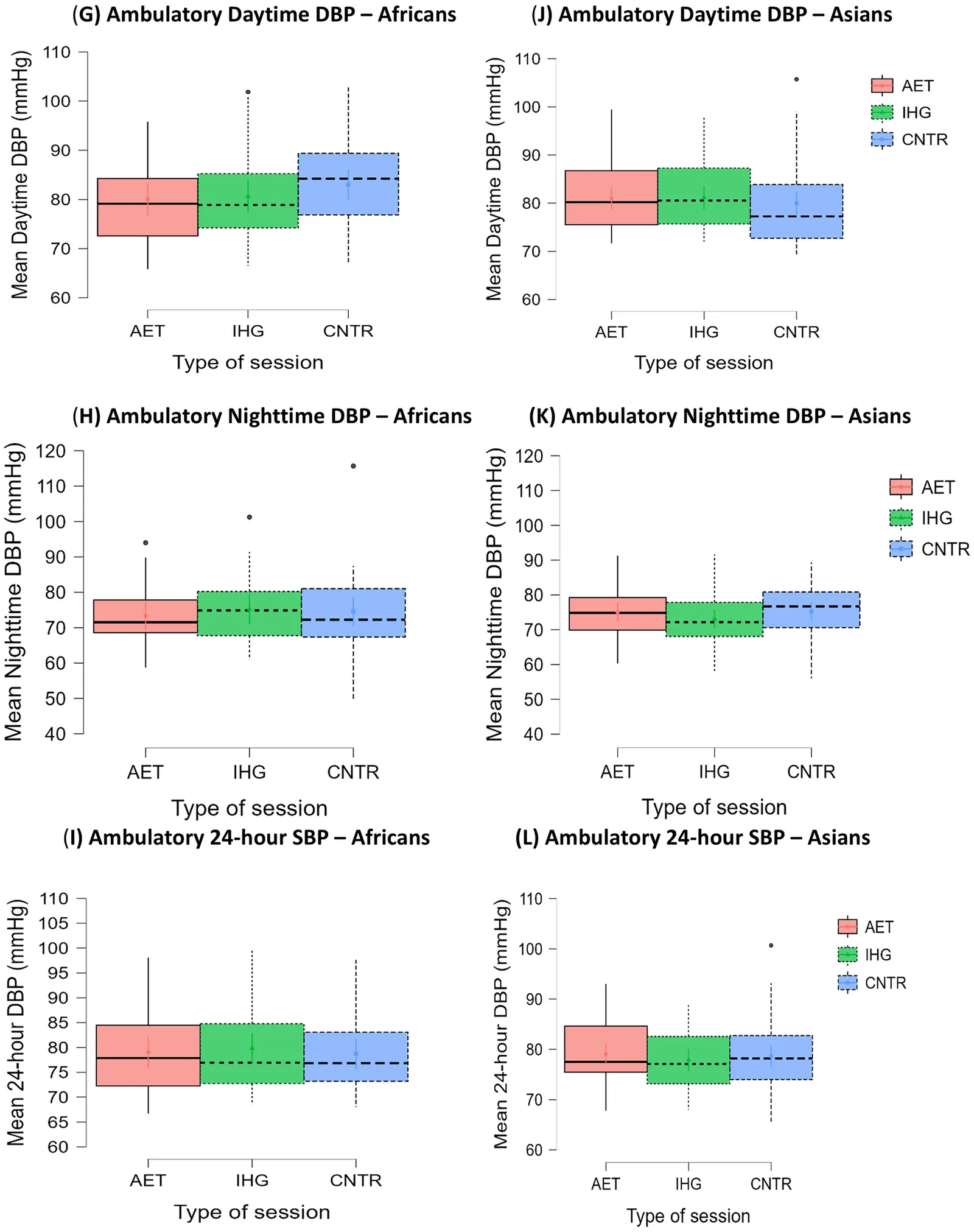
**(A–L)** average ambulatory daytime, nighttime, and 24-hour systolic and diastolic blood pressure values following aerobic exercise, isometric handgrip exercise, and control sessions in the African and south-Asian groups. Panels A–F present systolic blood pressure variables and panels G–L present diastolic blood pressure variables. Panels A–C and G–I represent the African group, while panels D–F and J–L represent the South-Asian group. ABP, ambulatory blood pressure; SBP, systolic blood pressure; DBP, diastolic blood pressure; AET, aerobic exercise training; IHG, isometric handgrip exercise; CNTR, control.

Figures 4A–L and supplementary Table S1 and S2 show data on ABP following the three experimental sessions for both ethnic groups. In comparison to the control session, daytime systolic and diastolic ABP showed clinically relevant, though statistically non-significant reductions following the aerobic exercise session [systolic ABP: −4.08, 95% CI: [−0.26, −7.75] mmHg, *p* = 0.08 and diastolic ABP: −3.07, 95% CI: [−0.27, −5.90] mmHg, *p* = 0.08] in individuals of African descent. In contrast, no reductions in daytime ABP were observed following the isometric handgrip session (see Figures 4A,G). No differences in daytime ABP were observed following a session of aerobic or isometric exercise compared with the control session in individuals of Asian descent (see Figures 4D,J).

The nighttime ABP was not affected in the individuals of African descent following an aerobic exercise session. Though, systolic nighttime ABP was significantly increased following an isometric exercise session [+4.74, 95% CI: (+1.13, +8.35), *p* = 0.03], compared to the control session (see Figure 4B). In individuals of Asian descent, a small but statistically non-significant in nighttime ABP was observed following the isometric exercise session [−4.76, 95% CI: [−8.57, 0.29], *p* = 0.11/−2.19, 95% CI: [−4.76, 0.38], *p* = 0.29] whereas no changes were observed after the aerobic exercise session (See Figures 4E,K).

Figures 3A–D. Systolic and diastolic BP at 30 min after the aerobic, isometric exercise or control sessions of the African group (*N* = 29) are presented in Figures 3A,B) and of the Asians (*n* = 28) in Figures 3C,D; * = *p* < 0.05 compared to control.

Figures 4A–L. Average ambulatory daytime, nighttime, and 24-hour systolic and diastolic blood pressure values following aerobic exercise, isometric handgrip exercise, and control sessions in the African and South-Asian groups. Panels A–F present systolic blood pressure variables and panels G–L present diastolic blood pressure variables. Panels A–C and G–I represent the African group, while panels D–F and J–L represent the South-Asian group. ABP, ambulatory blood pressure; SBP, systolic blood pressure; DBP, diastolic blood pressure; AET, aerobic exercise training; IHG, isometric handgrip exercise; CNTR, control.

### Postexercise hypotension: mean BP at hourly intervals for 8 h

The hourly mean BP for 8 h following aerobic and isometric exercise can be seen in Figure A–D and Supplementary Table S4 for the African and Asian group. When calculating the average change per hourly interval only the aerobic exercise session did lead to a non-significant reduction in the average change in the systolic BP over 8 h, equal to the daytime systolic ABP, [−4.08, 95% CI: (−0.26, −7.75) mmHg, *p* = 0.08] and a significant reduction in the average change in the diastolic BP over 8-hours, equal to the daytime diastolic ABP, [−3.09, 95% CI: (−0.27, −5.90) mmHg, *p* = 0.03] in the African group compared with the control group. No reduction was observed in the Asian group.

**Figure 5:**
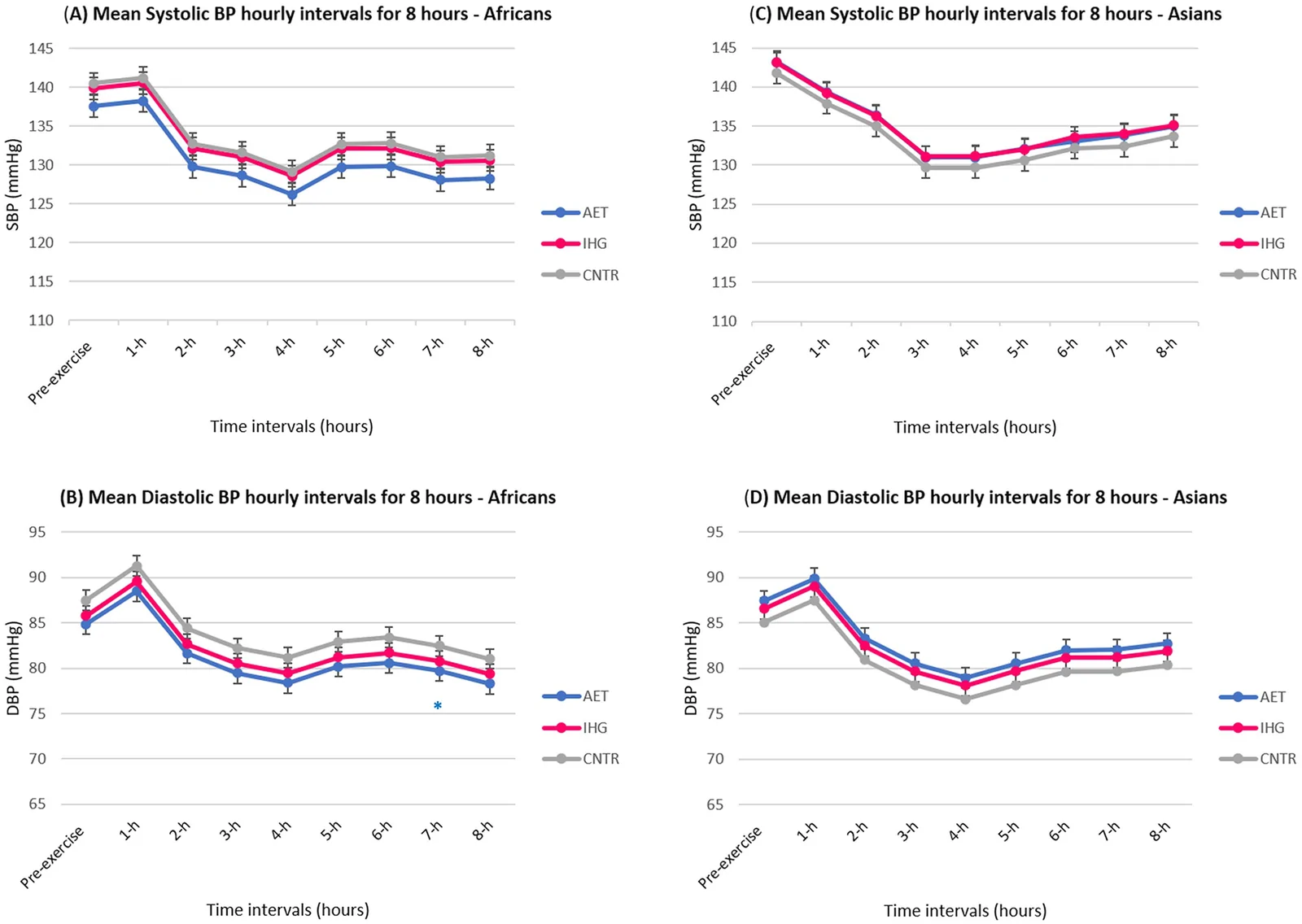
**(A–D)** mean systolic and diastolic BP at hourly intervals over 8 h following aerobic exercise, isometric handgrip exercise, and control sessions in the African group (panels A–B; *N* = 23) and the south-Asian group (panels C–D; *N* = 24). *P*-values were adjusted using Bonferroni correction.

Figures 5A–D. Mean systolic and diastolic BP at hourly intervals over 8 h following aerobic exercise, isometric handgrip exercise, and control sessions in the African group (Panels A–B; *N* = 23) and the South-Asian group (Panels C–D; *N* = 24). *P*-values were adjusted using Bonferroni correction.

## Discussion

This study is the first randomized controlled cross-over trial investigating PEH in a non-Caucasian Surinamese population, specifically including individuals of African and South-Asian descent with high normal BP or grade I hypertension. The main findings of this study showed that 1) aerobic exercise resulted in an immediate reduction in systolic BP at 30 min postexercise in both the African group (−9.03 mmHg, *p* < 0.001) and South-Asian group (−7.40 mmHg, *p* < 0.01), while diastolic BP was significantly reduced only in the South-Asian group (−4.41 mmHg, *p* = 0.01) and showed a non-significant reduction in the African group (−3.46 mmHg, *p* = 0.08); 2) a clinically relevant daytime PEH response after aerobic exercise was observed only in the African group, with a reduction in daytime systolic BP of −4.08 mmHg (*p* = 0.08) and daytime diastolic BP of −3.07 mmHg (*p* = 0.08); and 3) a single session of isometric handgrip exercise did not produce PEH in either the African or South-Asian group.

To date, only one study has investigated the immediate PEH response in individuals of African origin (30). In contrast to our findings, Ash et al. reported no change in BP after 20 min of moderate-intensity cycling in individuals of African descent with high-normal BP and stage I hypertension (30). The difference between our findings and those of Ash et al. may be explained by differences in exercise duration (30 vs. 20 min) and exercise modality (treadmill vs. cycling), as treadmill exercise involves a larger active muscle mass and may induce a greater hemodynamic stimulus. This explanation is consistent with Brasil et al., who reported that the magnitude and mechanisms of PEH may vary according to exercise prescription characteristics, including exercise modality, intensity, duration, and muscle mass involved (8).

In contrast to our findings, previous studies investigating the immediate PEH response in Asian populations have mainly included young normotensive individuals] (31–33), and reported no significant BP reductions 30 min postexercise. The absence of PEH observed in these previous studies is consistent with evidence showing that PEH is attenuated in individuals with normotension or lower baseline BP (16, 34). In addition to baseline BP, methodological differences, including small sample sizes, lower exercise stimuli, younger and physically active participants, and differences in exercise modality (cycling or brisk walking vs. treadmill walking), may also explain the absence of PEH. In contrast, the South-Asian participants in our current study consisted only of individuals with high—normal BP or stage 1 hypertension, with high baseline BP (138.17 ± 6.98/85.73 ± 4.57 mmHg) which could explain the immediate BP reductions.

The absence of immediate PEH following low-intensity isometric exercise in our two populations is consistent with three previous studies in hypertensive Caucasian populations using low- and moderate-intensity isometric exercise (35–37), although two studies have reported PEH 30 min after isometric exercise (16, 27, 38). Previous isometric exercise studies (37, 38) suggest that baseline BP may contribute to the BP response, as PEH has primarily been observed in participants with higher baseline BP values, such as 134/84 mmHg (27) and 135/77 mmHg (38). However, despite the presence of high baseline BP in our African population (142/88 mmHg) and Asian population (138/85 mmHg) PEH was still absent after isometric exercise and cannot be explained by baseline BP alone. Therefore, the absence of PEH in our study may reflect the variable acute BP response reported after a single session of isometric exercise rather than ethnic differences alone. Nevertheless, the available evidence remains inconsistent, as one previous study (38) showed that isometric exercise performed at different intensities and volumes did not reduce BP in hypertensive individuals. More recent evidence suggests that the BP-lowering effects of isometric exercise are more consistently observed after repeated training interventions rather than after a single exercise session (18, 19).

An unexpected finding was the increase in nighttime systolic BP following IHG in the African group. In contrast, previous studies evaluating ambulatory BP after acute isometric exercise have generally not reported increases in nighttime BP, including acute IHG (30) and isometric plank exercise (39). Although the exercise modality differed, these findings suggest that the type of isometric exercise may also influence the ambulatory BP response. Given the prognostic importance of nighttime BP (40), this isolated finding warrants further investigation.

Our findings in the daytime systolic and diastolic BP after an aerobic exercise session in the African group (−4.08 mmHg and −3.09 mmHg) are in line with the meta-analyses by Saco et al. (41), which reported reductions of −4.00 and −2.3 mmHg following aerobic exercise in a predominantly Caucasian population.. However, our findings in the daytime BP contrast with the earlier findings of Pescatello et al. (42) showing an increase in daytime BP and no change in daytime diastolic BP in normotensive and hypertensive African participants. One possible explanation for this contrasting finding is exercise-induced reductions in insulin, although this was not measured in the present study. Pescatello et al. (42) reported an association between reductions in insulin and decreases in daytime systolic and diastolic BP in African participants. This potential mechanism warrants further investigation. While we observed no effect on mean 24-hour BP in the African group, Pescatello et al. reported an increase in mean 19-hour systolic BP in Africans participants (43), whereas Saco et al. (41) reported a reduction in mean 24-hour systolic BP in predominantly Caucasian populations. Besides possible ethnic differences, heterogeneity in exercise volume and intensity, sample size, and definitions of daytime, nighttime, and 24-hour BP may also contribute to the variation in findings. Therefore, the literature on PEH remains inconclusive in the African population and warrants further investigation.

The literature on PEH and vasodilatory responses in African populations remains contradictory (44–47). While some studies suggest that Africans do not exhibit PEH following moderate-intensity aerobic exercise due to attenuated nitric oxide-mediated vasodilation (44), higher vascular resistance (48) and enhanced cardiovascular and sympathetic vasoconstrictor responses (49), Smith et al. (47) reported the opposite, namely greater vasodilatory capacity and dilatory reserve in Africans.

Interestingly, Pescatello et al. (45) found that variation in the endothelial nitric oxide synthase gene (NOS3) influenced PEH in African, but not in Caucasian participants, following vigorous-but not moderate-intensity aerobic exercise (45). Similarly, Pescatello et al. reported that the presence of multiple Angiotensinogen-Converting Enzyme (ACE) gene variants was associated with PEH in the Africans after vigorous,- but not moderate intensity aerobic exercise (43). Besides NOS3 and multiple ACE gene variants, Goessler et al. demonstrated that ACE polymorphisms can influence PEH following a single bout of aerobic exercise in Caucasian participants (50). Although NOS3 variation, multiple ACE gene variants, and ACE polymorphisms have been associated with PEH, whether these mechanisms contribute to the daytime PEH observed in our African participants remains unknown and warrants further investigation.

Our findings in the South-Asians strengthen the results of Chaturvedi et al., who reported an attenuated PEH response in South-Asians compared to Caucasians (51). However, Chaturvedi et al., evaluated PEH only immediately after exercise and not over a 24-hour period. Our study is the first trial to evaluate the acute 24-hour BP response to aerobic and isometric handgrip exercise in hypertensive South-Asians. South-Asians have a higher prevalence of insulin resistance, hyperglycemia and type 2 diabetes (52–55). Insulin resistance and hyperglycemia are associated with impaired sympathovagal activity and may contribute to an attenuated BP-lowering response to exercise (51). This mechanism may partly explain the absence of PEH in our South Asian population.

Another mechanism that may contribute to the absence of PEH is increased aortic stiffness in South-Asians due to hyperglycemia (51). Chaturvedi et al. showed that elevated aortic stiffness contributes to an attenuated PEH response independently of sympathetic activity. We therefore propose that hyperglycemia-related autonomic dysfunction and vascular stiffness may have contributed to the blunted PEH response in our South-Asian. Although not directly comparable to acute BP responses, Igarashi et al. reported chronic BP reductions after aerobic exercise in East-Asians (56). A recent publication provided insight into physiological differences between South- and East Asians that may influence BP responses to exercise (55). Compared with East-Asians, South-Asians have a less favorable metabolic profile, including greater insulin resistance, which may partly contribute to differences in BP responses (55). Whether these physiological differences explain the different BP responses observed between South- and East-Asians warrants further investigation.

Similar to the South-Asians, Africans also exhibit sympathetic overactivity at rest, which may contribute to increased vasoconstriction and higher BP. However, in contrast to South-Asians, despite similar resting sympathovagal activity, acute exercise in Africans seems to result in augmented sympathetic and hemodynamic responses (57). Recently, Bajdek et al. reported reduced vagal reactivation and increased sympathovagal balance following acute exercise sessions in young African women (58). However, the participants were normotensive and exercised at vigorous intensity. It is possible that exercise-induced improvements in autonomic function contributed to the favorable daytime systolic BP reductions observed in our African participants. More research is required to further acknowledge the role of the autonomic function in BP regulation following exercise in Africans population at different exercise intensities.

Our study includes several strengths and limitations. First, international guidelines (58) based on meta-analytic research performed in predominantly Caucasian populations recommend at least 30 min of moderate-intensity aerobic exercise (40%–60% HRR) on 5–7 days per week for individuals with hypertension (13, 14). The daily exercise recommendation aims to repeatedly induce PEH following a single aerobic exercise session. Furthermore, the guidelines advise the addition of resistance training 2–3 times per week (13, 14). In our study, we applied the recommended aerobic exercise prescription to investigate whether this can be translated into an effective antihypertensive tool to reduce BP in African and Asian populations and to evaluate the potential need for ethnic-specific exercise recommendations. Second, we used ambulatory BP monitoring measuring BP every 30 min over a 24-hour period, the gold standard for BP assessment (14), allowing evaluation beyond a limited number of postexercise measurements. Another strength was the randomized controlled cross-over design with the participants serving as their own control.

Our first limitation was that participants using antihypertensive medication were not excluded, because it was very difficult to find eligible participants not using antihypertensive medication, which would have resulted in an unrepresentative small sample size. Nevertheless, the inclusion of predominantly medicated hypertensive adults better reflects routine clinical practice. Another limitation is that ethnicity was classified according to grandparental ethnicity rather than genetic ancestry. Although this approach has previously been applied in Surinamese population research (20), genetic admixture cannot be excluded. Consequently, the proposed roles of NOS3 and ACE polymorphisms should be interpreted cautiously and require confirmation in studies incorporating genetic analyses. Although participants recorded their sleeping and waking times in a diary, sleep was not objectively verified, which should be considered when interpreting the nighttime BP findings. Lastly, our sample included 57 predominantly medicated adults of African (*N* = 29) and Asian origin (*N* = 28) with obesity, limiting our ability to conduct subgroup analyses according to sex, medication use, physically active, body weight, BP category, salt.

## Conclusion

In conclusion, a single session of aerobic exercise elicited a clinically relevant PEH response during the daytime period in individuals of African descent; however, this PEH response was not sustained over the 24-hour period. Furthermore, a single session of low-intensity isometric handgrip exercise did not induce a PEH response in the African or Asian populations and therefore warrants further investigation before it can be recommended as an antihypertensive exercise modality in these populations. Similarly, a single session of aerobic did not elicit PEH in the Asian population. The optimal BP-lowering effect of a single session of exercise in non-Caucasian populations may depend on factors such as exercise intensity, exercise modalities, exercise volume or frequencies, which should be further investigated.

## Data Availability

The original contributions presented in the study are included in the article/Supplementary Material, further inquiries can be directed to the corresponding author.
