# Supplementary material for: Effects of a single bout of low-intensity isometric or moderate-intensity aerobic exercise on postexercise hypotension in adults of African and south-Asian descent: a randomized controlled crossover trial
Source: Front Rehabil Sci. 2026 Jul 31;7:1882762. doi: 10.3389/fresc.2026.1882762 (PMC13501518; doi:10.3389/fresc.2026.1882762)
Supplement: Supplementary file 1 [file Table1.docx]

**Appendix**

*Supplementary Table S1. Systolic and diastolic blood pressure variables during the aerobic exercise, isometric handgrip exercise, and control sessions in the African group.*

| **BP variables** |  |  | **African descendants (N = 29)** | | |  |
| --- | --- | --- | --- | --- | --- | --- |
|  | N | AET | IHG | CNTR | Δ AET vs CNTR | Δ IHG vs CNTR |
| **Office BP** | | | | | | |
| Pre-intervention resting SBP (mmHg) | 29 | 139.58 ± 2.17 | 139.17 ± 2.17 | 138.41 ± 2.17 | +1.17 ± 1.93 (p=1.00) | +0.76 ± 1.93 (p=1.00) |
| Pre-intervention resting DBP (mmHg) | 29 | 85.84 ± 1.38 | 87.43 ± 1.38 | 85.77 ± 1.39 | +0.06 ± 1.22 (p=0.95) | +1.65 ± 1.22 (p=0.53) |
| Office SBP post-30-minute exercise (mmHg) | 29 | 136.54 ± 2.04 | 144.15 ± 2.04 | 145.58 ± 2.05 | **-**  **-9.03 ± 2.20*** **(p<0.001)** | -1.41 ± 2.20 (p=0.51) |
| Office DBP post-30-minute exercise (mmHg) | 29 | 86.21 ± 1.28 | 88.86 ± 1.29 | 89.68 ± 1.28 | -**3.46 ± 1.58** (p=0.08) | -0.81 ± 1.59 (p=0.60) |
| **Ambulatory BP** | | | | | | |
| Ambulatory daytime SBP (mmHg) | 23 | 128.23 ± 1.87 | 131.25 ± 1.83 | 132.31 ± 1.03 | **-4.08 ± 1.87** (p=0.08) | --1.06 ± 1.83 (p=1.00) |
| Ambulatory daytime DBP (mmHg) | 23 | 80.34 ± 1.60 | 81.12 ± 1.58 | 83.43 ± 1.56 | **-3.09 ± 1.44***  **(p=0.03)** | -2.30 ± 1.42  (p=0.10) |
| Ambulatory nighttime SBP (mmHg) | 27 | 120.61 ± 2.27 | 124.62 ± 2.33 | 119.87 ± 2.27 | +0.74 ± 1.77 (p=0.69) | **+4.75 ± 1.84***  (p=0.03) |
| Ambulatory nighttime DBP (mmHg) | 27 | 73.35 ± 1.93 | 75.15 ± 2.03 | 74.59 ± 1.93 | -1.24 ± 2.19 (p=1.00) | +0.56 ± 2.28 (p=1.00) |
| Ambulatory 24-hours SBP (mmHg) | 24 | 126.12 ± 1.98 | 129.03 ± 1.98 | 126.97 ± 1.97 | -0.85 ± 1.57 (p=0.53) | +2.06 ± 1.57 (p=0.57) |
| Ambulatory 24-hours DBP (mmHg) | 24 | 79.05 ± 1.51 | 79.80 ± 1.52 | 78.72 ± 1.51 | +0.33 ± 1.06 (p=1.00) | +1.08 ± 1.06 (p=0.92) |

**Table S1** Systolic and diastolic BP in the exercise and control sessions for pre-intervention, office BP, daytime, nighttime and 24 h monitoring for the African participants. Data are reported as estimated marginal means ± S.E., BP = blood pressure; AET = aerobic exercise training; IHG = isometric exercise training; CNTR = control; SBP = systolic blood pressure; DBP = diastolic blood pressure. P-values are adjusted using Bonferroni adjustment.

*Supplementary Table S2. Supplementary Table S2. Systolic and diastolic blood pressure variables during the aerobic exercise, isometric handgrip exercise, and control sessions in the South-Asian group.*

| **BP variables** |  |  | **Asian descendants (N = 28)** | | |  |
| --- | --- | --- | --- | --- | --- | --- |
|  | N | AET | IHG | CNTR | Δ AET vs CNTR | Δ IHG vs CNTR |
| **Office BP** | | | | | | |
| Pre-intervention resting SBP (mmHg) | 28 | 141.28 ± 2.40 | 142.16 ± 2.40 | 140.27 ± 2.41 | +1.00 ± 1.92 (p=1.00) | +1.87 ± 1.90 (p=0.97) |
| Pre-intervention resting DBP (mmHg) | 28 | 86.69 ± 1.66 | 86.35 ± 1.66 | 85.14 ± 1.66 | +1.55 ± 1.37 (p=0.77) | +1.21 ± 1.27 (p=0.77) |
| Office SBP post-30-minute exercise (mmHg) | 28 | 136.78 ±78 | 144.03 ± 1.89 | 143.19 ± 1.86 | -**7.40 ± 2.44***  (p<0.01) | +0.19 ± 2.45 (p=0.93) |
| Office DBP post-30-minute exercise (mmHg) | 28 | 84.96 ± 1.21 | 89.46 ± 1.23 | 89.52 ± 1.22 | **-4.41 ± 1.57*** (p=0.01) | -0.25 ± 1.56 (p=0.87) |
| **Ambulatory BP** | | | | | | |
| Ambulatory daytime SBP (mmHg) | 24 | 132.83 ± 1.96 | 133.98 ± 1.96 | 133.21 ± 1.94 | -0.38 ± 1.60 (p=1.00) | +0.78 ± 1.65 (p=1.00) |
| Ambulatory daytime DBP (mmHg) | 24 | 81.25 ± 1.20 | 81.59 ± 1.20 | 80.68 ± 1.19 | +0.57 ± 1.00  (p=1.00) | +0.91 ± 0.98 (p=1.00) |
| Ambulatory nighttime SBP (mmHg) | 25 | 126.26 ± 2.26 | 122.60 ± 2.29 | 127.04 ± 2.24 | -0.78 ± 2.07 (p=0.70) | -4.43 ± 2.11 (p=0.11) |
| Ambulatory nighttime DBP (mmHg) | 25 | 75.07 ± 1.26 | 73.11 ± 1.28 | 75.31 ± 1.25 | -0.23 ± 1.29 (p=0.85) | -2.19 ± 1.31 (p=0.28) |
| Ambulatory 24-hours SBP (mmHg) | 27 | 131.32 ± 1.74 | 128.63 ± 1.76 | 131.24 ± 1.71 | +0.08 ± 1.46 (p=0.95) | -2.60 ± 1.48 (p=0.23) |
| Ambulatory 24-hours DBP (mmHg) | 27 | 79.23 ± 1.05 | 78.14 ± 1.06 | 78.98 ± 1.04 | +0.24 ± 0.81 (p=0.76) | -0.84 ± 0.81 (p=0.60) |

**Table S2** Systolic and diastolic BP in the exercise and control sessions for pre-intervention, office BP, daytime, nighttime and 24 h monitoring for the Asian participants. Data are reported as estimated marginal means ± S.E., BP = blood pressure; AET = aerobic exercise training; IHG = isometric exercise training; CNTR = control; SBP = systolic blood pressure; DBP = diastolic blood pressure. P-values are adjusted using Bonferroni adjustment.

*Supplementary Table S3.* Mean ambulatory BP changes calculated over the course of 1 – 8 hours following aerobic exercise and isometric handgrip exercise compared to the control session in the African and South-Asian groups.

| Mean BP changes | **African group (N=23)** | | | | **Asian group (N = 24)** | | | |
| --- | --- | --- | --- | --- | --- | --- | --- | --- |
|  | AET vs CNTR | p-value | IHG vs CNTR | p-value | AET vs CNTR | p-value | IHG vs CNTR | p-value |
| Mean ambulatory 1-hour SBP (mmHg) | -1.00 ± 3.20 | 1.00 | -1.56 ± 3.26 | 1.00 | +1.25 ± 2.81 | 1.00 | +2.83± 2.82 | 0.95 |
| Mean ambulatory 1-hour DBP (mmHg) | -1.42 ± 2.56 | 1.00 | -3.18 ± 2.64 | 0.69 | +3.04 ± 2.26 | 0.53 | +1.95 ± 2.27 | 1.00 |
| Mean ambulatory 2-hours SBP (mmHg) | -5.27 ± 2.95 | 0.22 | -2.63 ± 3.00 | 1.00 | +0.40 ± 2.55 | 1.00 | +1.45 ± 2.55 | 1.00 |
| Mean ambulatory 2-hours DBP (mmHg) | -3.77 ± 2.28 | 0.29 | -2.63 ± 2.23 | 0.77 | +3.18 ± 1.77 | 0.21 | +2.54 ± 1.76 | 0.45 |
| Mean ambulatory 3-hours SBP (mmHg) | -3.49 ± 3.11 | 0.78 | -3.04 ± 2.06 | 0.95 | -0.05 ± 2.19 | 1.00 | +2.14 ± 2.20 | 1.00 |
| Mean ambulatory 3-hours DBP (mmHg) | -2.89 ± 1.99 | 0.44 | -3.11 ± 1.97 | 0.34 | +2.86 ± 1.58 | 0.21 | +2.67 ±1.57 | 0.27 |
| Mean ambulatory 4-hours SBP (mmHg) | -3.57 ± 2.47 | 0.44 | -3.03 ± 2.42 | 0.63 | +0.38 ± 1.92 | 1.00 | +1.38 ± 1.23 | 1.00 |
| Mean ambulatory 4-hours DBP (mmHg) | -1.89 ± 1.74 | 0.83 | -2.95 ± 1.71 | 0.25 | +3.41 ± 1.35 | 0.03* | +3.03 ± 1.34 | 0.07 |
| Mean ambulatory 5-hours SBP (mmHg) | -3.78 ± 2.21 | 0.28 | -2.01 ± 2.21 | 1.00 | -1.24 ± 1.84 | 1.00 | +0.65 ± 1.85 | 1.00 |
| Mean ambulatory 5-hours DBP (mmHg) | -1.74 ± 1.66 | 0.88 | -2.81 ± 1.64 | 0.25 | +1.87± 1.24 | 0.40 | +1.96 ± 1.23 | 0.33 |
| Mean ambulatory 6-hours SBP (mmHg) | - 3.78 ± 2.13 | 0.22 | -1.67 ± 2.09 | 0.66 | +0.93 ± 1.79 | 1.00 | +0.83 ± 1.79 | 1.00 |
| Mean ambulatory 6-hours DBP (mmHg) | -2.40 ± 1.60 | 0.40 | -2.93 ± 1.57 | 0.20 | +1.48 ± 1.14 | 0.59 | +1.51 ± 1.13 | 0.55 |
| Mean ambulatory 7-hours SBP (mmHg) | -4.24 ± 2.01 | 0.10 | -1.71 ± 1.97 | 1.00 | -0.53 ± 1.69 | 1.00 | +0.61 ± 1.69 | 1.00 |
| Mean ambulatory 7-hours DBP (mmHg) | -2.96 ± 1.44 | 0.14 | -2.78 ± 1.48 | 0.18^#^ | +0.95 ± 0.99 | 1.00 | +1.56 ± 0.98 | 0.71 |
| Mean ambulatory 8-hours SBP (mmHg) | **-4.08 ± 1.87** | 0.08 | -1.05 ± 1.82 | 1.00 | -0.38 ± 1.65 | 1.00 | +0.78 ± 1.65 | 1.00 |
| Mean ambulatory 8-hours DBP (mmHg) | **-3.09 ± 1.44** | **0.03*** | -2.30 ± 1.42 | 0.10 | +0.57 ± 1.00 | 1.00 | +0.91 ± 0.98 | 1.00 |

**Table S3.** Systolic and diastolic BP average change over a period of 1 to 8 hours in the African and Asian group. Data are reported as mean± SE, minus (-) = reduction; plus (+) = increase; AET = aerobic exercise training; IHG = isometric handgrip exercise; CNTR = control; p-value adjusted using Bonferroni adjustment

*Supplementary Table S4.* Hourly means of BP over 1 – 8 hours after exercise compared to control following aerobic exercise and isometric handgrip exercise compared to the control session in the African and South-Asian groups. .

| Mean BP changes | **African group (N=23)** | | | | **Asian group (N = 24)** | | | |
| --- | --- | --- | --- | --- | --- | --- | --- | --- |
|  | AET vs CNTR | p-value | IHG vs CNTR | p-value | AET vs CNTR | p-value | IHG vs CNTR | p-value |
| Mean 1-hour SBP (mmHg) | -3.36 ± 3.22 | 0.89 | -1.61 ± 3.28 | 1.00 | +1.48 ± 3.19 | 1.00 | -1.20 ± 2.28 | 1.00 |
| Mean 1-hour DBP (mmHg) | -1.71 ± 2.50 | 1.00 | -1.87 ± 258 | 1.00 | +3.04 ± 2.26 | 0.53 | +1.95 ± 2.27 | 1.00 |
| Mean 2-hours SBP (mmHg) | -4.55 ± 2.80 | 0.31 | -2.73 ± 2.85 | 1.00 | +0.61 ± 2.68 | 1.00 | +0.24 ± 2.64 | 1.00 |
| Mean 2-hours DBP (mmHg) | -3.94 ± 2.16 | 0.20 | -3.60 ± 2.20 | 0.30 | +3.18 ± 1.77 | 0.21 | +2.54 ± 1.76 | 0.45 |
| Mean 3-hours SBP (mmHg) | -1.74 ± 3.51 | 1.00 | -0.65 ± 3.37 | 1.00 | -1.54 ± 2.68 | 1.00 | -1.01 ± 2.72 | 1.00 |
| Mean 3-hours DBP (mmHg) | -1.82 ± 2.42 | 1.00 | +1.22 ± 2.3 | 1.00 | +1.43 ± 2.21 | 1.00 | +2.19 ± 2.22 | 0.98 |
| Mean 4-hours SBP (mmHg) | -4.26 ± 3.11 | 0.51 | -3.02 ± 2.98 | 0.93 | +0.38 ± 1.92 | 1.00 | +1.38 ± 1.23 | 1.00 |
| Mean 4-hours DBP (mmHg) | -1.52 ± 2.64 | 1.00 | -2.59 ± 2.55 | 0.93 | +2.94 ± 1.1.95 | 0.40 | +3.43 ± 1.94 | 0.23 |
| Mean 5-hours SBP (mmHg) | -2.56 ± 2.73 | 1.00 | -0.97 ± 275 | 1.00 | -1.24 ± 1.84 | 1.00 | +0.65 ± 1.85 | 1.00 |
| Mean 5-hours DBP (mmHg) | -1.39 ± 2.34 | 1.00 | -2.12 ± 2.37 | 1.00 | -2.77± 2.29 | 0.68 | -1.37 ± 2.18 | 1.00 |
| Mean 6-hours SBP (mmHg) | -4.32 ± 2.82 | 0.37 | -0.20± 2.82 | 1.00 | +0.93 ± 1.79 | 1.00 | +0.83 ± 1.79 | 1.00 |
| Mean 6-hours DBP (mmHg) | **-5.65 ± 2.31** | **0.04*** | -3.74 ± 2.31 | 0.31 | -0.68 ± 2.16 | 1.00 | -1.27 ± 2.15 | 1.00 |
| Mean 7-hours SBP (mmHg) | **-5.77 ± 2.57** | **0.07** | -2.64 ± 2.61 | 0.94 | -0.53 ± 1.69 | 1.00 | +0.61 ± 1.69 | 1.00 |
| Mean 7-hours DBP (mmHg) | **-6.04 ± 2.05** | **0.01^*^** | -2.87 ± 2.07 | 0.50 | -0.16 ± 2.05 | 1.00 | -0.01 ± 2.04 | 1.00 |
| Mean 8-hours SBP (mmHg) | -2.09 ± 2.17 | 1.00 | +1.76 ± 2.17 | 1.00 | -0.38 ± 1.65 | 1.00 | +0.78 ± 1.65 | 1.00 |
| Mean 8-hours DBP (mmHg) | **-4.45 ± 2.62** | **0.08** | -1.81 ± 2.62 | 0.49 | -1.90 ± 2.29 | 1.00 | +0.38 ± 2.36 | 1.00 |

**Table S4.** Systolic and diastolic BP at hourly intervals over 1 to 8 hours in the African and Asian group. Data are reported as mean± SE, minus (-) = reduction; plus (+) = increase; AET = aerobic exercise training; IHG = isometric handgrip exercise; CNTR = control; p-value adjusted using Bonferroni adjustment; * = p-value <0.05
